# Acute stress reveals different impacts in male and female *Zdhhc7*-deficient mice

**DOI:** 10.1007/s00429-021-02275-y

**Published:** 2021-04-20

**Authors:** Nicole Kerkenberg, Christa Hohoff, Mingyue Zhang, Ilona Lang, Christiane Schettler, Evgeni Ponimaskin, Lydia Wachsmuth, Cornelius Faber, Bernhard T. Baune, Weiqi Zhang

**Affiliations:** 1 Department of Mental Health, University of Münster, Münster, Germany; 2 Otto Creutzfeldt Center for Cognitive and Behavioral Neuroscience, University of Münster, Münster, Germany; 3 Cellular Neurophysiology, Hannover Medical School, Hannover, Germany; 4 Clinic of Radiology, University of Münster, Münster, Germany; 5 Department of Psychiatry, Melbourne Medical School, University of Melbourne, Melbourne, VIC Australia; 6 Florey Institute for Neuroscience and Mental Health, University of Melbourne, Melbourne, VIC Australia

**Keywords:** Palmitoylation, *Zdhhc7*-deficiency, Acute stress, Behavior, Small animal imaging, Eletrophysiological recordings

## Abstract

Numerous processes of neuronal development and synaptic plasticity in the brain rely on the palmitoyl acyltransferase ZDHHC7, as it palmitoylates various synaptic and extrasynaptic proteins such as neural cell adhesion molecule (NCAM) or gamma-aminobutyric acid (GABA_A_) receptors. In addition, ZDHHC7 palmitoylates sex steroid hormone receptors and is, therefore, indirectly linked to mental disorders that often occur because of or in conjunction with stress. In this work, we investigated how ZDHHC7 affects stress responses in mice. For this purpose, genetically modified mice with a knockout of the *Zdhhc7* gene (KO) and wild-type (WT) littermates of both sexes were exposed to acute stressors or control conditions and examined with regard to their behavior, brain microstructure, gene expression, and synaptic plasticity. While no behavioral effects of acute stress were found, we did find that acute stress caused reduced mRNA levels of *Esr1* and *Esr2* coding for estrogen receptor α and β in the medial prefrontal cortex of male WT and KO mice. Strikingly, after acute stress only male KO mice showed reduced mean fiber lengths of the medioventral hippocampus. Furthermore, *Zdhhc7*-deficiency impaired synaptic plasticity in mice of both sexes, while acute stress improved it in females, but not in male mice. Taken together, our findings suggest that ZDHHC7 plays a modulatory role in the brain that leads to sex-specific stress responses, possibly due to estrogen receptor-mediated signaling pathways.

**Supplementary Information:**

The online version contains supplementary material available at 10.1007/s00429-021-02275-y.

## Introduction

The most common lipid modification involved in the trafficking and function of proteins in neurons is S-palmitoylation (Fukata and Fukata 2010), a process catalyzed by palmitoyl acyltransferases (PATs), which reversibly attach a 16-C fatty acid to their target proteins. This leads to a conformational change in transmembrane proteins, regulating their activity and interaction with other proteins (Fukata and Fukata 2010; Shipston 2011). Importantly, a key conserved PAT involved in various synaptic and extrasynaptic proteins is the protein ZDHHC7. Among other targets, ZDHHC7 palmitoylates neural cell adhesion molecule (NCAM) (Ponimaskin et al. 2008; Fukata and Fukata 2010), whereby this modification is required for neurite outgrowth of hippocampal neurons (Ponimaskin et al. 2008). In addition, ZDHHC7 also palmitoylates gamma-aminobutyric acid (GABA_A_) receptors (Naumenko and Ponimaskin 2018), which are major mediators of fast synaptic inhibition in the mammalian brain (Rathenberg et al. 2004) and are involved in anxiety and object recognition memory (Naumenko and Ponimaskin 2018).

Furthermore, ZDHHC7 and its paralog ZDHHC21 palmitoylate the sex steroid receptors for estrogen (ERα, ERβ), progesterone (PR), and androgen (AR) (Pedram et al. 2012). These receptors are of particular interest since ERs are involved in fear, anxiety and depression because they maintain serotonergic neurons (Suzuki et al. 2013), while PRs have a broader role in regulating cognition, mood, neurogenesis or myelination (Brinton et al. 2008). Further, estrogen- and androgen-mediated synaptic plasticity in the hippocampus is critical for maintaining healthy memory processes (Ooishi et al. 2012). Notably, sex steroid hormones can exert their effects not only via nuclear receptors but also via palmitoylation of receptors that are localized on the plasma membrane (Norman et al. 2004; Pedram et al. 2012; Baudry et al. 2013).

These non-genomic response receptors allow for rapid signal transmission (Baudry et al. 2013) and influence the electrophysiological properties of neurons within milliseconds to minutes across the nervous system (Baudry et al. 2013). Further, rapid signaling by ERs and PRs also affects intracellular signaling cascades, downstream gene expression, and neuroanatomical structure (Balthazart and Ball 2006; Ooishi et al. 2012; Baudry et al. 2013). Rapid ER-mediated signaling, for example, is crucial for early hippocampus organization in a sex-specific manner (Meitzen et al. 2017). Thereby, ERα interacts with metabotropic glutamate receptors (mGluR) within caveolin 1 (CAV1)-generated microdomains at the plasma membrane and mediates rapid estradiol effects (e.g., Boulware et al. 2005; Meitzen et al. 2012). Although ZDHHC7 and ZDHHC21 are both responsible for rapid ER signaling (Meitzen et al. 2013), only the expression of ZDHHC7 differs at specific developmental stages in a sex-specific manner (Meitzen et al. 2017).

Furthermore, the sex steroid hormone receptors are represented in brain regions such as the prefrontal cortex (PFC), hippocampus, thalamus (Marroco and McEwan 2016) and amygdala (Kudwa et al. 2009), which belong to the cortico-limbic system (Baudry et al. 2013; Seney and Sibille 2014). These regions play an important role in the function of memory and emotion (Bandler et al. 2000) and are particularly sensitive to sex differences and stress (Seney and Sibille 2014). Hippocampal cells are known to have a sex-specific susceptibility to stress (Shors et al. 2001, 2004; Koss and Frick 2017), and the hippocampus has also been found to show sex-dependent modifications in gene expression (Marrocco et al. 2017) as well as a particular sensitivity to stress (Conrad 2008). Importantly, the hippocampus plays a critical role in learning and memory formation, and morphological disturbances in the hippocampus could lead to cognitive deficits (Sapolsky 2001).

Another particularly stress-sensitive region is the medial PFC (mPFC) (Lehmann et al. 2017), which is also influenced by biological sex (Drzewiecki et al. 2016). Therefore, the stress in combination with changes in sex steroid hormone signaling pathways may alter the functional connectivity of the highly interconnected mPFC (Kumar et al. 2013) and, thus, affect mPFC-related emotional, socio-affective and visceromotor outcomes (Bandler et al. 2000; Damasio et al. 2000; Uylings et al. 2003; Franklin and Chudasama 2012). In turn, sex steroid hormone receptors could, among other palmitoylated proteins, influence the neuronal structure and connectivity of brain regions both in the physiology and in the pathophysiology (Zagni et al. 2016).

In this respect, ZDHHC7 appears to be important for regulating multiple neuronal functions with regard to stress and sex differences in the brain. In fact, our recent data demonstrated that *Zdhhc7*-deficiency affected the brain microstructure, synaptic plasticity and behavior in mice (Hohoff et al. 2019). In that study, *ex-vivo* diffusion tensor imaging (DTI) of mice with *Zdhhc7*-knockout (KO) revealed altered hippocampal fiber structures in male and female mice. In particular, fibers of the hippocampal medioventral CA were shorter in KO mice compared to wild types (WT) and remained within the medioventral area, while the fibers of WT mice projected roughly towards the mPFC. Furthermore, *Zdhhc7*-deficiency impaired the long-term potentiation (LTP) in hippocampal synaptic plasticity at Schaffer collateral CA1 synapses in both male and female mice, and it increased excitatory glutamatergic synaptic transmission in the prelimbic mPFC of females but decreased it in males. Moreover, locomotion was increased and anxiety-related behavior was decreased exclusively in *Zddhc7*-KO females (Hohoff et al. 2019). Together, this and other previous studies raise important questions on how *Zdhhc7* alters the stress response in male and female mice.

To directly test this, in the current study we exposed adolescent *Zdhhc7* mutants of both sexes to an acute stressor and characterized their responses using behavioral tests, DTI, gene expression analysis, and electrophysiology. We hypothesized that acute stress would affect *Zdhhc7*-deficiency-mediated phenotypes in a sex-specific manner.

## Materials and methods

### Experimental animals

For the experiments, *Zdhhc7*-WT and -KO mice were used (Hohoff et al. 2019). The animals were bred in the central animal facility (ZTE) of the University of Münster and weaned between postnatal weeks 4–5. Same-sex littermate pairs (KO and WT) were housed in Makrolon Type II L cages (37 × 21 × 14 cm^3^) with sawdust as bedding material and access to food and water ad libitum.

The present work was in accordance with all current regulations covering animal experimentation in Germany and the EU (European Communities Council Directive 2010/63/EU). All procedures as well as the specific study presented here were in accordance with the National Animal Welfare Law approved by the responsible government authority (Landesamt für Natur, Umwelt und Verbraucherschutz Nordrhein-Westfalen, LANUV-NRW, Germany: Az84-02.04.2016.A416). All efforts were made to minimize animal suffering as well as to reduce the number of animals used in this study to a minimum necessary for reliable statistical analyses.

### Experimental design

At the age of 6 weeks, KO and WT littermate pairs (one WT-KO littermate per cage) of both sexes were randomly assigned to the control non-stress group (female WT: *n* = 11; female KO: *n* = 11; male WT: *n* = 11; male KO: *n* = 11) or to the experimental acute-stress group (female WT: *n* = 10; female KO: *n* = 10; male WT: *n* = 9; male KO: *n* = 9). After a 2-week habituation phase in the experimental housing room, the experimental group was exposed to an acute stress paradigm, while the control group was kept under control conditions in their home cages. The acute stressor represented restraint stress and included a locomotive restriction (in a 50 ml tube with air holes) with tones (80 dB "white noise") in a soundproof chamber for 60 min.

At the age of 9 weeks, all animals (82 mice) underwent behavioral tests, which included the measurement of anxiety-like behavior and locomotion in the elevated plus-maze (EPM) test, a basal spatial working memory test via the spontaneous alternation test (SAT), a social exploration test via the social interaction test (SI), and a general stress and attention test using the nest building test (NB). In the following week, cognitive function was measured using the object recognition test (ORT), and depression-like behavior was assessed using the tail suspension test (TS, details see below).

**Fig. 1:**
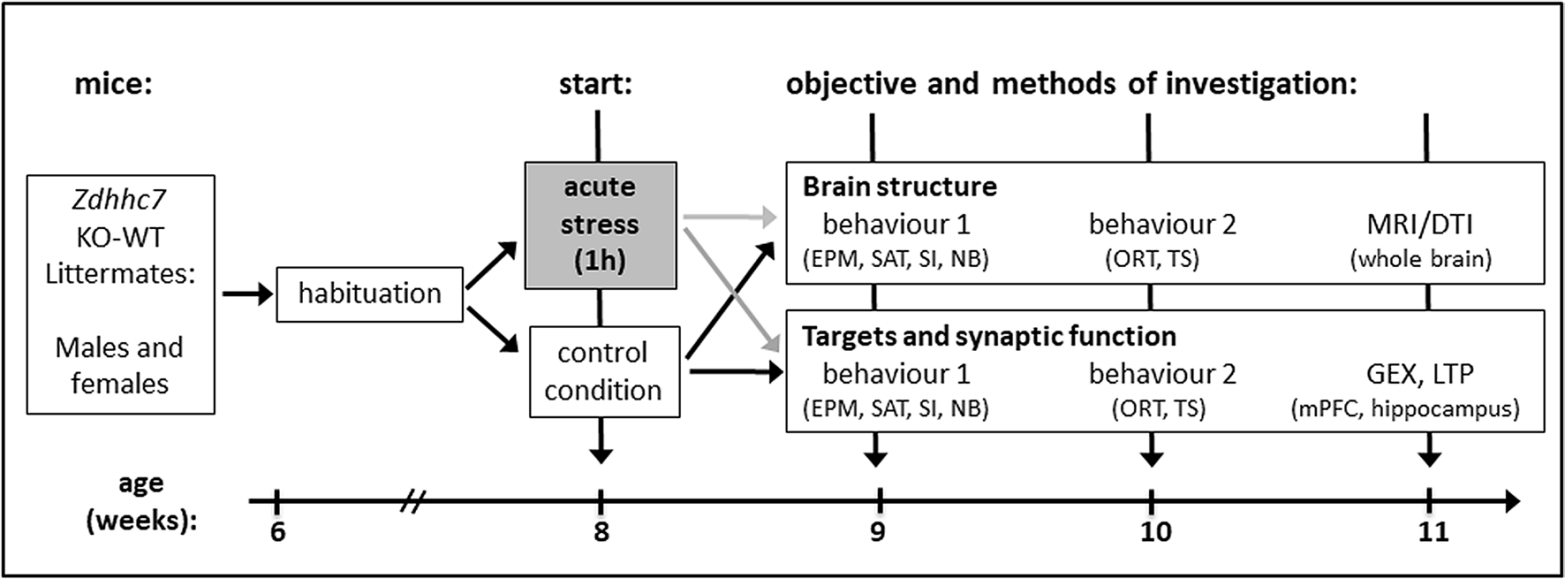
Time schedule illustrating the acute stress paradigm. *Zdhhc7* knockout (KO) and wild-type (WT) littermates underwent 2 weeks of habituation and were exposed to acute stress or control conditions at 8 weeks of age, followed by 2 weeks of behavioral testing using the elevated plus-maze test (EPM), spontaneous alternation test (SAT), social interaction test (SI), nest building test (NB), object recognition test (ORT) and tail suspension test (TS). At the age of 11 weeks, animals were sacrificed and the whole brain of the first subsample was used for brain structural analysis (using magnetic resonance imaging (MRI)-based diffusion tensor imaging (DTI), while in the second subgroup, the medial prefrontal cortex (mPFC) was used for gene expression (GEX), and the hippocampus was used for electrophysiological long-term potentiation (LTP) analyses

At the age of 11 weeks, the animals were sacrificed, and biological material was collected either for DTI fiber tractography measurements (excised whole brains of 32 animals) or for gene expression analysis (mPFC) and electrophysiological LTP measurements (hippocampus; complete brains of 50 animals). Unfortunately, it was not possible to evaluate material for gene expression and LTP from every animal whose behavior was analyzed, because of technical problems with these advanced methods (n = 10 animals excluded). An overview of the complete experimental design can be found in Fig. 1.

### Behavioral testing

A total of 82 mice (9–11 per group) was behaviorally tested in the experimental housing room by the same experimenter. The subsequent behavioral analysis was carried out for all tests (EPM, SAT, SI, NB, ORT, TS) using ANY-maze software (Stoelting).

The EPM was performed in an apparatus (illuminated with 70 lx) consisting of four arms originating from a center, in which two opposite arms were surrounded by walls while the other two arms were open. The arms of the EPM, each 45 cm long and 5 cm wide, were 50 cm above the floor. At the beginning of the 5-min test, the animal was placed in the center zone with its head directed toward a closed arm to freely explore the maze. To evaluate anxiety-like behavior, we measured the relative amount of time spent on open arms (Handley and Mithani 1984; Walf and Frye 2007).

The SAT was performed in a Y-shaped apparatus whose three identical arms (A, B and C) were surrounded by walls, and each of them originated from a common center. The animals were placed into one arm and could freely move in the apparatus for 6 min (illuminated with 50 lx). Correct alternation was characterized by exploring all three different arms one after the other in any order. The percentage of alternations was calculated as described by Kim et al. (2007) as an index for spatial working memory.

The SI was performed in an open-field apparatus (42 × 42 × 40 cm; illuminated with 50 lx) and consisted of two trials of 2.5 min each. In the first trial, the mouse was placed in the right corner of the box where it could freely explore the apparatus and a small empty cage, positioned at one wall. After that, the test mouse was returned to its home cage while an unfamiliar same-sex “target” mouse was placed into the small cage. In the second trial, the test mouse could freely explore again but in the presence of the target mouse. The social interaction ratio (SI ratio) was calculated as described by Kim et al. (2017).

In the NB test, each mouse was placed alone in a fresh cage with clean bedding and a nest pad (pressed cotton of about 2.6 g) for 24 h. The type of nest pad was familiar to the animals since birth as a common nesting material. The built nest was photographed after 1, 3, and 7 h (light period) and after 24 h (after a complete night period), and the process of nesting was classified according to the so-called Deacon score from 1 to 5 (Deacon, 2006).

The ORT was performed in an open-field apparatus (42 × 42 × 40 cm; illuminated with 50 lx) and consisted of four trials of 5 min each, three on the first day and one on the second day. The test started with a habituation trial where the mouse was placed in the middle of the front wall and was free to explore the empty apparatus. After this, the mouse was returned to its home cage for 5 min. In the second trial, the object exploration trial, the mouse could explore two identical novel objects (A + A) placed in the middle of the apparatus. In the third trial, 60 min after the second, one object A was replaced by a novel, unfamiliar object B located at the exact same position as object A was before (A + B). After 24 h, on the next day, object B was replaced by another novel, unfamiliar object C (A + C). Mice were considered to be exploring when they touched or sniffed the object. To measure cognitive function, the amount of time spent exploring the novel object in relation to the total time spent exploring both objects was used to calculate the percentage of recognition for short-term memory (second trial) and long-term memory (third trial) (Hohoff et al. 2019).

In the TS test, the animal was hung upside down from a rod by means of an adhesive strip attached to the mouse’s tail (2 cm from the tail end). A 5 cm-long plastic cylinder over the tail of the animals prevented them from pulling up along their tail or the adhesive tape. In addition, care was taken to ensure that the mice could not hold on to the walls. During the 6-min test period, we observed whether the animals actively tried to free themselves from their position or behaved inactively.

### Neuroimaging (MRI-based DTI fiber tractography)

A subgroup of 32 mice (*n* = 4 per group) was used for MRI-based DTI fiber tractography according to Hohoff et al. (2019). Briefly, mice were deeply anesthetized, perfused with PBS and 4% PFA, and their brains were dissected. After 4 days of incubation in 4% PFA, brains were washed and afterwards incubated in contrast agent solution (2 mmol/L Magnevist, Bayer Pharma AG, Berlin, Germany) for 1 day. Brains were scanned with a 9.4 T small animal imaging system after they were embedded into small plastic tubes in 1% agar enriched with contrast agent (2 mmol/L Magnevist). The DTI&Fiber Tool (Kreher et al. 2006; https://www.uniklinik-freiburg.de/mr-en/research-groups/diffperf/fibertools.html) was used to perform DTI fiber tractography. We, therefore, used the same region of interest (ROI) as Hohoff et al. (2019) for the left and right medioventral hippocampus, which was based on the Allen Mouse Brain Connectivity Atlas (2011) (Oh et al. 2014; http://connectivity.brain-map.org/). ROI-based Mori fiber tracking was then used to determine fiber statistics for the medioventral hippocampal CA. We determined the fractional anisotropy (FA) values within the ROIs as well as the fiber lengths and fiber numbers.

### Gene expression analysis

Another subset of 50 mice was used for gene expression analysis of the mPFC. Due to technical problems, the RNA of 10 animals could not be extracted in sufficient quantity and quality, resulting in sample sizes of 4–6 per group (female control WT: *n* = 4; female control KO: *n* = 4; female stressed WT: *n* = 6; female stressed KO: *n* = 6; male control WT: *n* = 5; male control KO: *n* = 5; male stressed WT: *n* = 5; male stressed KO: *n* = 5). Mice were deeply anesthetized and decapitated, then left and right mPFC were dissected and stored in RNAlater. For RNA extraction, RNAlater was discarded from the tissue and probes were transferred into a ZR BashingBead™ Lysis Tube (mixed 0.5 mm and 0.1 mm, Zymo Research, USA) with 800 µl trizol and homogenized using a BeadBug microtube homogenizer for 30 s at maximum speed (4000 rpm). RNA was extracted from the supernatant using the Direct-zol™ RNA MiniPrep Plus Kit (Zymo Research, USA) according to the manufacturer's instructions with minor modifications: To ensure a sufficient removal of DNA, we extended DNAse I treatment (30 min, 37 °C). In addition, 15 µl glycogen (1 µg/µl) was added as a co-precipitant to enhance RNA concentration. Initial RNA quality and concentration were measured using a photometer (Eppendorf, Germany) resulting in an average concentration of 46.3 ng/μl.

Subsequent cDNA synthesis was performed using the High Capacity cDNA Reverse Transcription Kit (Thermo Fischer Scientific, Germany) according to the manufacturer's specifications with 200 ng total RNA (20 ng/μl) of each sample.

The qPCRs were performed using TaqMan® Gene Expression Assays with TaqMan™ Gene Expression Master Mix (both: ThermoFisher Scientific, Germany) according to the manufacturer's instructions. The mRNAs selected for analysis were estrogen receptor α (*Esr1*) (assay ID: Mm00433149_m1), estrogen receptor β (*Esr2*) (assay ID: Mm00599821_m1), progesterone receptor (*Pgr)* (assay ID: Mm00435628_m1), androgen receptor (*Ar*) (assay ID: Mm00442688_m1), Caveolin 1 (*Cav1*) (assay ID: Mm00483057_m1) and Caveolin 3 (*Cav3*) (assay ID: Mm01182635_m1). The phosphoglycerate kinase 1 (*Pgk1*) mRNA (assay ID: Mm00435617_m1) was used as an internal control to normalize samples with different endogenous quantities. All qRT-PCR assays were performed in triplicate, and relative expression levels were calculated using the comparative C_t_ method (Livak and Schmittgen, 2001). Statistical analyses were performed using the $\Delta C_{{\text{t}}}$ values. The significant effects of stress and sex were illustrated by fold change values:

$$\Delta C_{{\text{t}}} = \, C_{{{\text{t }}({\text{Target}})}} {-} \, C_{{{\text{t }}({\text{Internal control}})}}$$

$$\Delta \Delta C_{{\text{t}}} = \, \Delta C_{{{\text{t }}({\text{Target}})}} {-} \, \Delta C_{{{\text{t }}({\text{Calibrator}})}}$$

$${\text{Fold change }} = \, 2^{{ - \, \Delta \Delta C_{{\text{t}}} }} .$$

To calculate the $\Delta \Delta C_{{\text{t}}}$ value, the mean value of the controls or the females served as calibrator. The fold change was always relative to the expression of the control group (control/female). It should be noted that normalization over one's own mean always leads to a value of approx. 1. We calibrated to females to show the sex effect compared to males. For the stress effect, we calibrated to the controls to illustrate the difference to the acute stressed animals. For comparison, all gene expression results were illustrated in Suppl. Fig. 3 with $\Delta C_{{\text{t}}}$ values.

### Electrophysiological recordings

The subset of 50 mice that was used for gene expression was also used for electrophysiological recordings in hippocampal slices of both hemispheres. Again, 10 animals had to be excluded due to technical problems during the measurements, resulting in sample sizes of *n* = 5 per group. Slice preparation and electrophysiological recordings were performed as described previously (Agarwal et al. 2014; Saffari et al. 2016; Wehr et al. 2017). Briefly, acute transverse hippocampus slices (300 μm) were cut in ice-cold oxygenated preparation solution (3 mM KCl, 1.25 mM NaH_2_PO_4_, 6 mM MgSO_4_, 26 mM NaHCO_3_, 0.2 mM CaCl_2_, 10 mM glucose, 218 mM sucrose). Afterward, slices were incubated for 1 h with 32 °C warm oxygenated artificial cerebrospinal fluid (ACSF; 126 mM NaCl, 3 mM KCl, 1.25 mM NaH_2_PO_4_, 1 mM MgSO_4_, 26 mM NaHCO_3_, 2 mM CaCl_2_, 10 mM glucose, aerated with 95% O_2_ and 5% CO_2_ (3–4 ml/min). For the extracellular recordings, Schaffer collaterals were stimulated with an electrode placed in the stratum radiatum at the CA3/CA1 junction as described by Agarwal et al. (2014). The magnitude of fEPSPs was measured as amplitude (baseline to peak) and slope (20–80% level of the falling phase). Baseline fEPSCs were set to about 50% of the maximum responses. LTP was induced by three trains separated by 20 s, each train consisting of 100 Hz stimulation for 1 s. Post-train responses were measured every 20 s for 60 min. fEPSPs were filtered by a four-pole Bessel filter at a corner frequency of 2 kHz and digitized at a sampling rate of 20 kHz using the DigiData 1400A interface (Molecular Devices, Sunnyvale, CA). Responses to extracellular stimulation were then recorded in the stratum radiatum in the CA1 region to study short-term potentiation (STP), determined as the average of responses in the first 5 min after high-frequency stimulation (HFS), and LTP, which was determined as the average of response in the last 10 min.

### Statistical analyses

All statistical tests were computed using SPSS (version 26, IBM Corp., Ehningen, Germany) or Prism (Version 8.0, GraphPad Software, San Diego, CA, USA). Datasets were checked for normal distribution by one-sample Shapiro–Wilk tests. Behavioral data were analyzed by a parametric repeated measures ANOVA using WT-KO littermates as paired samples and sex and stress as between-subject factors. Explorative comparison of only two groups was done using a Mann–Whitney *U* test (K vs. AS; F vs. M) or a Wilcoxon sign rank test (WT vs. KO) for non-parametric testing of DTI data, gene expression data, and electrophysiology data.

## Results

### Impact of genotype and sex on behavior

**Fig. 2:**
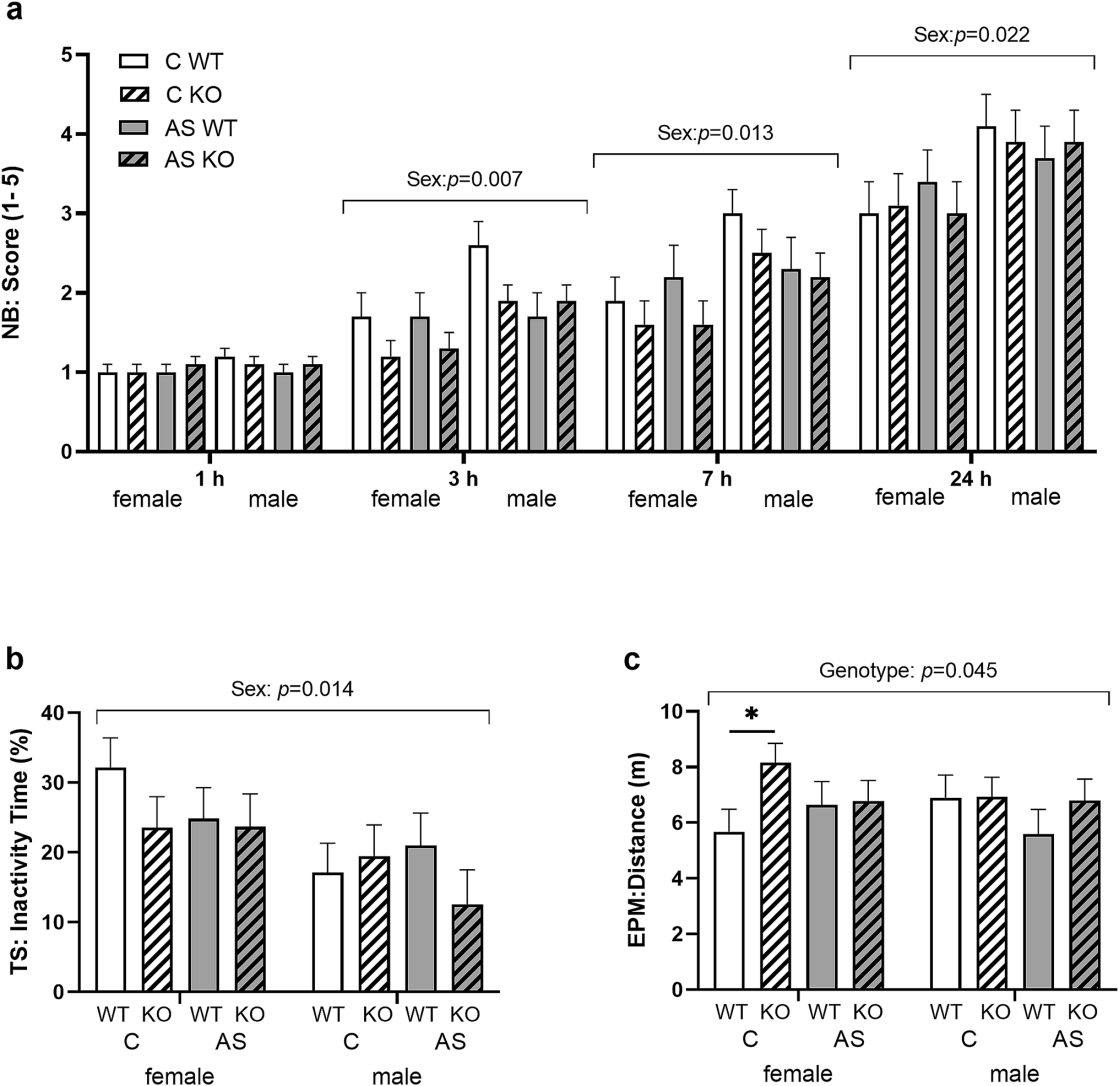
Effects of sex on nest-building (**a**) and depression-like behavior (**b**) and effects of *Zdhhc7*-deficiency on locomotion (**c**) of *Zdhhc7* knockout (KO) and wild-type (WT) mice under control conditions (C) or acute stress (AS). **a** Females showed decreased nest-building (NB) behavior after 3, 7 and 24 h compared to males. **b** Females showed increased depression-like behavior in the tail-suspension test (TS) compared to males. **c** *Zdhhc7* KO females showed increased locomotor activity as indicated by the total distance traveled in elevated plus-maze (EPM). Bars present group means (± SEM) asterisks depict the level of significance (*p < 0.05). Sample sizes were *n* = 9–11 per group

To investigate the effect of *Zdhhc7*-deficiency in combination with acute restraint stress, mice underwent a set of behavioral tests. Animals of both sexes were analyzed regarding general stress load, locomotion, anxiety-like and depression-like behaviors as well as social exploration and cognitive function. We found that male mice reached higher nesting scores than females in the NB test (Fig. 2a), which revealed significant sex effects after 3 h (*F*_(1,37)_ = 8.099, *p* = 0.007), 7 h (*F*_(1,37)_ = 6.792, *p* = 0.013), and 24 h (*F*_(1,37)_ = 5.686, *p* = 0.022). This suggests that females, in general, had a higher stress load in the test situation than males. With regard to locomotion, as indicated by alternation between the EPM arms, KO mice traveled a longer distance than WTs (*F*_(1,37)_ = 4.303, *p* = 0.045, Fig. 2b). This effect of genotype was confirmed by post-hoc dependent Wilcoxon sign rank tests. Here, female KOs showed significantly more locomotion than their WT littermates under control conditions (*z* = − 1.956, *p* = 0.050). No significant effects of *Zdhhc7* genotype, sex and acute stress were found for anxiety-like behavior, regarding time spent on open arms (*p*_all_ > 0.05, Online Resource Suppl. Fig. 1a, b). For depression-like behavior in the TS test, we found a significant effect of sex (*F*_(1,37)_ = 6.595, *p* = 0.014). Here, females spent more time inactive and, thus, exhibited more depression-like behavior than males (Fig. 2c). In contrast, genotype and stress did not have a significant effect on the inactivity of the animals (*p* > 0.05). In addition, no differences were observed regarding sociability in the SI, spatial working memory in the SAT, as well as short- and long-term memory in the ORT (*p*_all_ > 0.05, Online Resource Suppl. Fig. 1c–f). Although there were relatively few changes in the above-mentioned behavioral domains, we found that *Zdhhc7*-deficiency affects locomotion in females. General sex differences were detected in NB and TS tests, suggesting higher general stress load and depression-like behavior exclusively in female mice.

### Acute stress resulted in reduced mean fiber lengths only in male KO mice

**Fig. 3:**
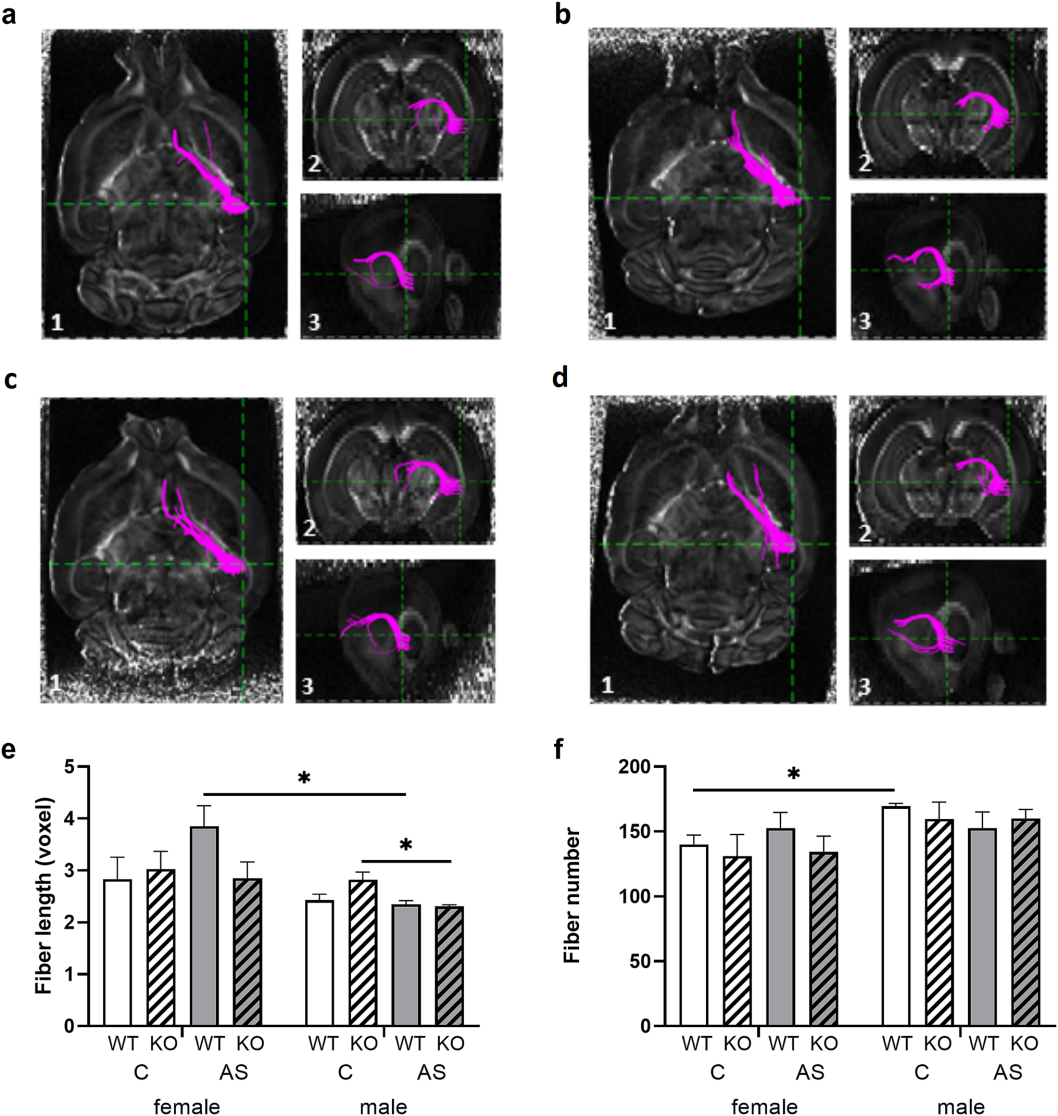
Differential effects of *Zdhhc7*-deficiency on the hippocampal microstructure of *Zdhhc7*-knockout (KO) and wild-type (WT) mice under control conditions (C) or acute stress (AS) **a–d** DTI&Fiber Tool-based images of fixated mouse brains to obtain Mori fiber tracking data used to analyze structural connectivity between medioventral hippocampal CA and medial prefrontal cortex (mPFC). **a** Illustrates a control *Zdhhc7-*WT mouse compared to **b** a control *Zdhhc7*-KO mouse, while **c** illustrates a acute stressed *Zdhhc7-*WT mouse compared to **d** a stressed *Zdhhc7*-KO mouse all in the different imaging planes axial (1), coronal (2), and sagittal (3). **e, f** Corresponding fiber statistics of the medioventral hippocampal CA. **e** Mean fiber length was significantly reduced in stressed KO males compared to control KO males, as well as stressed WT males compared to stressed WT females. **f** Fiber number was significantly higher in male WT controls compared to female WT controls. Bars represent group means (± SEM), and asterisks depict level of significance *p < 0.05. Sample sizes were *n* = 4 per group

We further used MRI-based DTI fiber tractography to test whether *Zdhhc7*-mediated rapid signaling pathways affect the neuroanatomical structure of the hippocampal medioventral CA region in a sex- and stress-dependent manner (Fig. 3a–d). Acute stress significantly reduced the mean fiber length in the right medioventral hippocampus of male KO mice compared to controls (*U* = 0.00, *z* = − 2.309, *p* = 0.029, Fig. 3e). Furthermore, sex differences in fiber lengths were found between stressed WT females and stressed WT males, while mean fiber lengths were significantly lower in males (*U* = 16.00, *z* = 2.309, *p* = 0.029, Fig. 3e). A sex effect was also observed in fiber numbers between control WTs. Here, the data revealed higher fiber numbers in males compared to females (*U* = 0.00, *z* = 2.323, *p* = 0.029, Fig. 3f). However, no effects of *Zdhhc7*-deficiency on fiber lengths, fiber numbers, or FA values were found in both left and right medioventral hippocampus (*p*_all_ > 0.05, Online Resource Suppl. Fig. 2). Altogether, male WT control mice showed higher fiber numbers than females, while acute stress impaired hippocampal medioventral CA in *Zdhhc7*-deficent males but not females. Furthermore, in WTs stress leads to lower fiber lengths in males compared to females.

### Acute stress resulted in lower gene expression in male mice

**Fig. 4:**
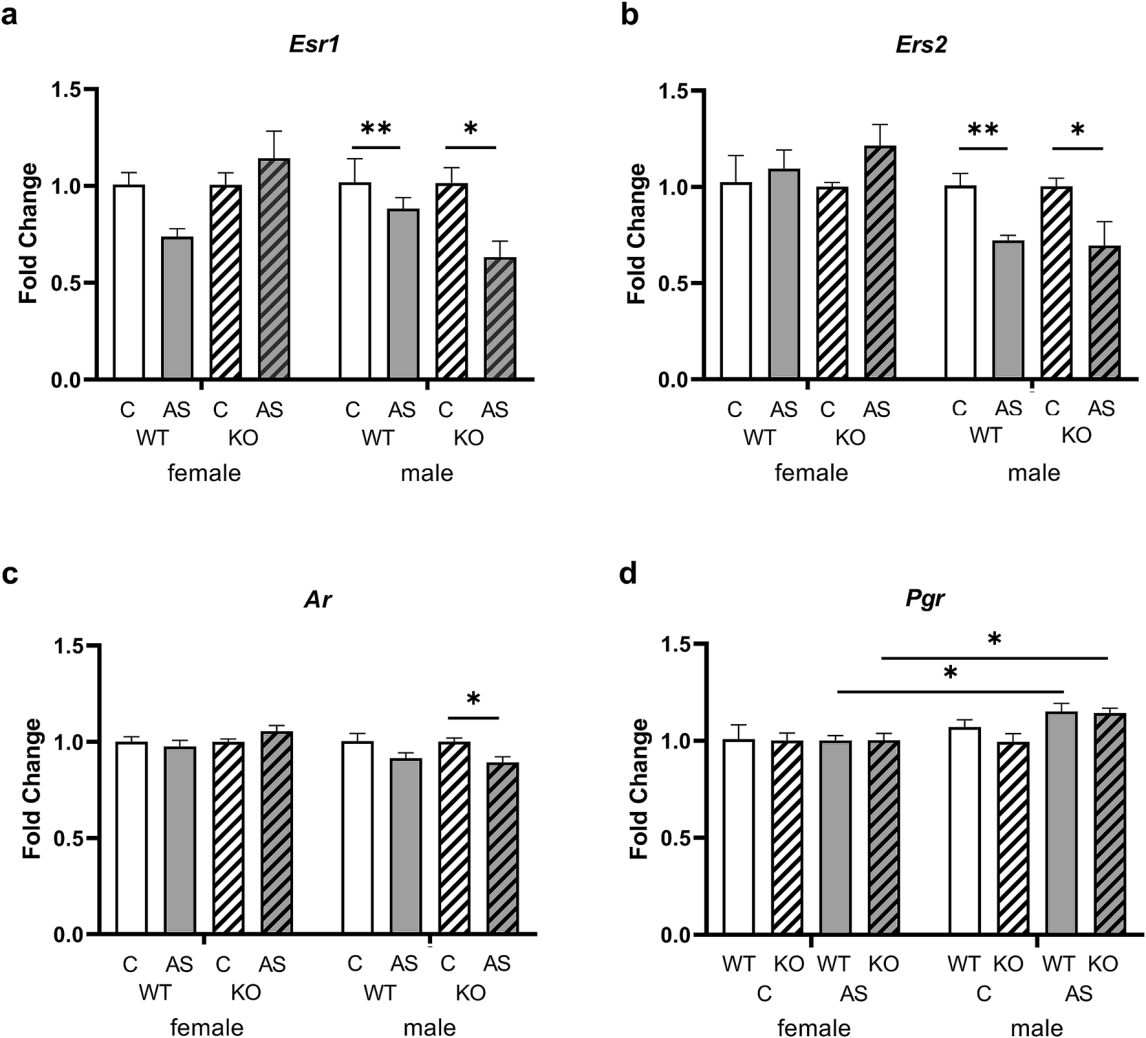
Relative expression (fold change) of candidate genes of *Zdhhc7*-knockout (KO) and wild-type (WT) mice under control conditions (C) or acute stress (AS). **a**–**d** Gene expression of estrogen receptor α (*Esr1)* (**a**), estrogen receptor β (*Esr2)* (**b**), and androgen receptor (*Ar)* (**c**) in the left medial prefrontal cortex (mPFC) with fold change calibrated to the controls to show the difference regarding acute stress according to the significant stress effects. Gene expression of progesterone receptor (*Pgr)* (**d**) in the right mPFC with fold change calibrated to females to show the difference regarding males according to the significant sex effects. Bars represent group means (± SEM) and asterisks depict level of significance *p < 0.05. Sample sizes were *n* = 4–6 per group

The possible effects of genotype, stress, and sex on downstream gene expression of the left and right mPFC was analyzed using quantitative RT-PCR. In all findings, stress led to lower gene expression in *Zdhhc7*-deficent males compared to control males, whereas no stress effects were detectable in females (*p*_all_ > 0.05). Effects of stress were found only in males for the gene expression of *Esr1* (male WT: *U* = 25.00, *z* = 2.611, *p* = 0.008; male KO: *U* = 24.00, *z* = 2.402, *p* = 0.016, Fig. 4a), *Esr2* (male WT: *U* = 25.00, *z* = 2.611, *p* = 0.008; male KO: *U* = 23.00, *z* = 2.193, *p* = 0.032, Fig. 4b), and *Ar* (male KO: *U* = 23.00, *z* = 2.193, *p* = 0.032, Fig. 4c) in the left mPFC but not in the right mPFC (*p*_*all*_ > 0.05). Sex differences, indicating higher gene expression in control (i.e., non-stressed) males compared to control females, were found for KOs in *Esr2* (control KO: *U* = 20.00, *z* = – 2.449, *p* = 0.016, Online Resource Suppl. Fig. 3b) and *Ar* (control KO: *U* = 20.00, *z* = – 2.449, *p* = 0.016, Online Resource Suppl. Fig. 3d). In contrast, *Ers1* was less expressed in stressed KO males compared to stressed KO females (stressed KO: *U* = 3.00, *z* = – 2.191, *p* = 0.030, Online Resource Suppl. Fig. 3a).

The only gene for which expression showed significant effects for sex (males vs. female) in the right (WT, AS: *U* = 29.00, *z* = 2.556, *p* = 0.009; KO, AS: *U* = 28.00, *z* = 2.373, *p* = 0.017, Fig. 4d) but not in the left mPFC (*p* > 0.05) was *Pgr*. Here, male WT and KO mice expressed more *Pgr* after acute stress than females. Again, no genotype effects could be observed in the left and right mPFC (*p*_all_ > 0.05, Online Resource Suppl. Fig. 3). Looking at the fold changes of the respective genes, however, it is noticeable that there were only slight deviations between the groups (Fig. 4a–d). As we only analyzed the expression of the above-mentioned genes, no statement can be made about the extent to which the expressed receptors were post-translationally palmitoylated by ZDHHC7.

### Acute stress improves *Zdhhc7*-deficiency-induced impairments of synaptic plasticity exclusively in females

**Fig. 5:**
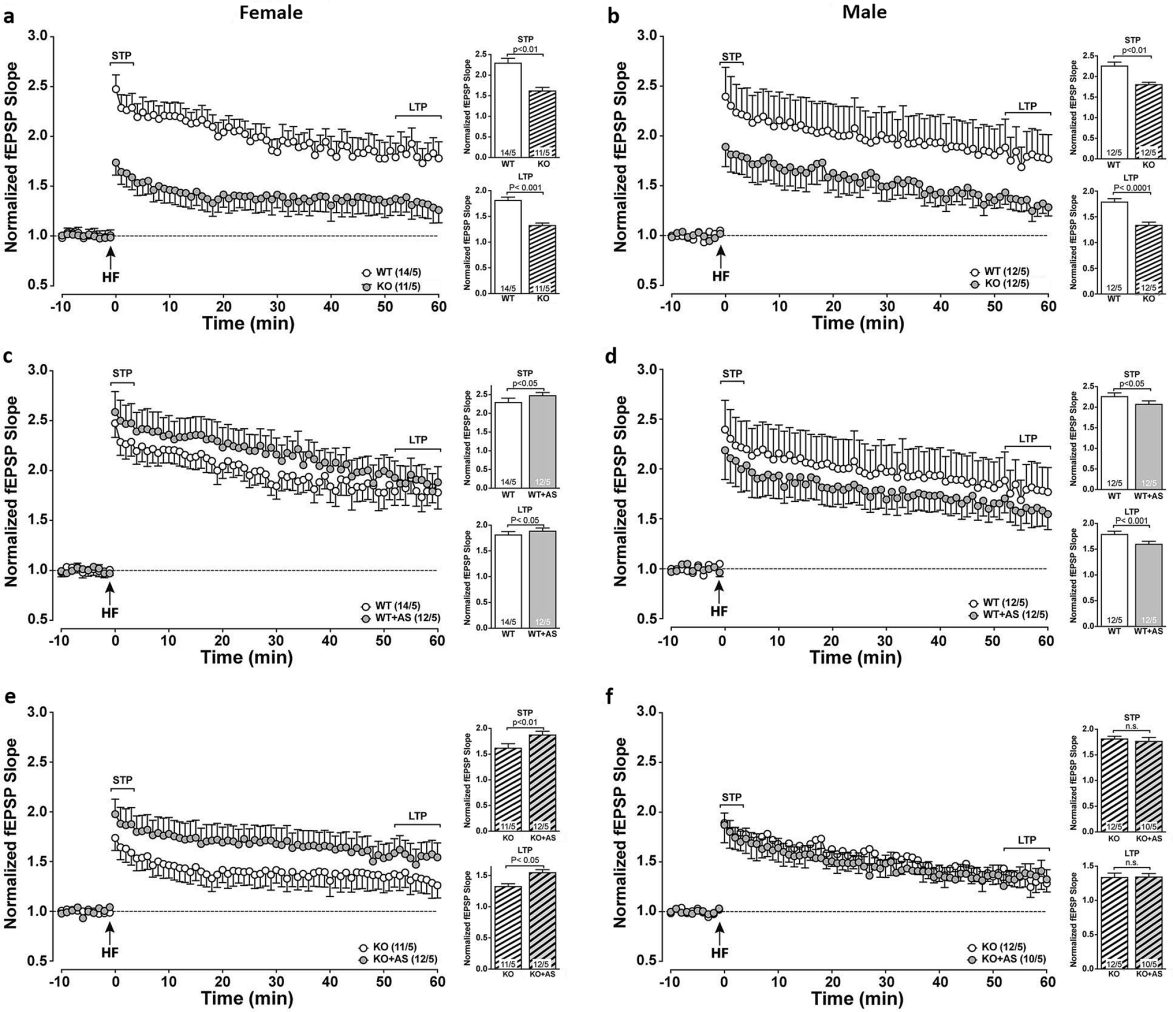
Long-term potentiation (LTP) of *Zdhhc7*-knockout (KO) and wild-type (WT) mice under control conditions (C) or acute stress (AS) **a**, **b** LTP at the SC-CA1 synapse in female (**a**) and male (**b**) WT and KO mice in control conditions. Insets show sample traces of responses before and after high-frequency stimulation (HFS). Slopes of spontaneous excitatory postsynaptic currents (fEPSP) were normalized to baseline and plotted against time. Time point 0 (arrow) represents HFS application. Short-term potentiation (STP) and LTP magnitude and were significantly impaired in control KO mice when compared to WT mice of both sexes. **c**, **d** LTP at the SC-CA1 synapse in WT control mice compared to stressed female (**c**) and male (**d**) WT mice. Acute stress significantly improved STP and LTP magnitude in WT females but reduced it in WT males. Insets show sample traces of responses before and after HFS. **e**, **f** STP and LTP magnitude were significantly improved in stressed KO females compared to control KO females, while no differences could be found between stressed and control males. Insets show sample traces of responses before and after HFS. Bars represent group means (± SEM), with the bottom numbers indicating sample sizes per group (*n*/*N*: recordings/total number of animals)

Subsequently, we tested the hippocampal STP and LTP induced at Schaffer collateral CA1 synapses in each sex. In all groups, both the input–output curves as well as the paired-pulse facilitation were unaltered, suggesting that there were no genotype-, stress-, or sex-specific changes in basal excitability (Online Resource Suppl. Fig. 4). As shown in Fig. 5a, b, LTP was significantly decreased in both female KO mice (Fig. 5a, STP: *U* = 25.00, z = 2.507, *p* = 0.007; LTP: *t* = 22.7, *df* = 16, *p* < 0.001) and male KO mice (Fig. 5b, STP: *U* = 25.00, *z* = 2.506, *p* = 0.007; LTP: *t* = 18.4, *df* = 18, *p* < 0.0001) compared to WT mice under control conditions. We further studied the effect of acute stress on each sex and observed that in females, there was an increase in LTP in the hippocampal slices of stressed WT mice compared to WT controls (Fig. 5c, STP: *U* = 25.00, *z* = – 1,99, *p* = 0.039; LTP: *t* = 3.1, *df* = 18 *p* = 0.005), whereas in stressed WT males, the LTP was suppressed in contrast to control WT males (Fig. 5d, STP: *U* = 25.00, *z* = 2.506, *p* = 0.012; LTP *t* = 8.0, *df* = 18, *p* < 0.0001). In *Zdhhc7*-KO mice, we observed that LTP was increased in female *Zdhhc7* KOs under stress condition compared to female KO controls (Fig. 5e, STP: *U* = 0.00, *z* = – 2.506, *p* = 0.012; LTP: *t* = 12.7, *df* = 18, *p* < 0.0001). However, in KO males LTP was not altered by acute stress compared to in control KO males (Fig. 5f, STP: *U* = 19.00, *z* = 1.250, *p* = 0.210; LTP: *t* = 0.283, *df* = 18, *p* = 0.783). Collectively, these data show that acute stress improved the STP and LTP in the hippocampus of both female WT and KO mice, while it impaired them in male WT mice and had no effect on male KO mice.

## Discussion

Our data demonstrated for the first time that even a short stress paradigm of 1 h duration leads to sex-specific stress responses in brain structure and function as well as in gene expression in the *Zdhhc7* mouse model. In the current study, sex-specific differences were found with regard to nest-building behavior, depression-like behavior and locomotion (Fig. 2). These results are in line with the current state of research, suggesting that single housing during the NB test could be more distressing for females than for males (Mackintosh 1981). Further, the data show that young females typically display more depression-like behavior than males (Walf et al. 2009; Frye 2011). Increased locomotion in KO females was also found in our recent study and might point to an explorative, disinhibited behavior induced by the lack of *Zdhhc7* (Hohoff et al. 2019).

Since palmitoylation of NCAM has been shown to be most strongly stimulated by ZDHHC7 (Ponimaskin et al. 2008), we initially assumed that deficiency of *Zdhhc7* could possibly lead to a reduction of NCAM-stimulated neurite outgrowth and, thus, to a reduction or shortening of fibers in the hippocampus. Surprisingly, our present data did not support this, since no effect of *Zdhhc7* on fiber length or number was found in KO compared to WT mice. Little is known about the effect of acute stress on brain microstructure, but the hippocampal neurons are known to be particularly sensitive to chronic stress (Conrad 2008), and the susceptibility of hippocampal cells to chronic stress differs between sexes (Shores et al. 2001, 2004; Koss and Frick 2017). It has further been shown that chronic stress causes atrophy of dendrites and dendritic spines in the pyramidal neurons of the hippocampal CA3 area and suppresses neurogenesis of dentate gyrus granule neurons (Bennett 2011). Therefore, stress-induced suppression of neurogenesis in the dentate gyrus could possibly also affect other hippocampal regions and their connections. Although the effect of chronic stress on hippocampal neurons has already been demonstrated (Shores et al. 2001, 2004; Conrad 2008; Bennett 2011; Koss and Frick 2017), in the current study we could now show that even acute short stress influences the hippocampal microstructure.

In our MRI-DTI analysis, the fiber numbers of male WTs were higher compared to female WTs under control conditions (Fig. 3f). However, after acute stress, the male WT mice had reduced mean fiber lengths compared to those in female WT mice. Moreover, stress led to reduced fiber lengths in *Zdhhc7*-deficient males compared to control KO males (Fig. 3e). Thus, the present results demonstrate that *Zdhhc7*-deficiency might lead to a higher stress vulnerability in male mice, such that even a short stressor could possibly lead to a reduction of dendrites or/and reduce neurogenesis. As shown by Hemmer et al. (2014), induced neural stem cells that were implanted into the dentate gyrus migrated to CA1, CA2 and CA3 regions and connected to the corresponding local networks within a few weeks. It is quite possible that in the current study, reduced neurogenesis could also have led to shortened mean fiber lengths two weeks after the stressor.

As a part of the cortico-limbic network, the mPFC is particularly sensitive to environmental stressors, and its organization is further influenced by biological sex (Drzewiecki et al. 2016). Additionally, estrogen receptors have been shown to act sex specifically in stress-mediated behaviors (Georgiou et al. 2019). Furthermore, female mice show a more robust HPA axis response during and after stressors compared to males (Oyola and Handa 2017). In the present study, acute stress led to reduced levels of sex steroid receptor mRNAs in the left mPFC exclusively in control males (Fig. 4a–c; WT: *Esr1*, *Esr2*; KO: *Esr1*, *Esr2*, *Ar*) and reduced *Esr1* expression in KO males. Such sex-specific stress responses were also found in the right mPFC after acute stress, resulting in reduced expression of *Pgr* in males (Fig. 4d; WT and KO) as compared to females, which might result from higher circulating estradiol levels in females. The fact that the stress effects on RNA levels did not translate into behavioral effects could have been because of the relative low fold change differences of all analyzed genes. An effect of *Zdhhc7*-deficiency on gene expression was not found in this study. Furthermore, we cannot say whether lower mRNA levels are associated with lower palmitoylation of the expressed receptors. Therefore, we can only speculate on the extent to which the rapid signaling pathways of sex steroid hormone receptors are affected by *Zdhhc7* knockout.

In the brain, ZDHHC7 is sex-specifically regulated with CAV1 (Meitzen et al. 2017). As previous studies have suggested that glutamatergic transmission in adult brains depends on a functional ZDHHC7/CAV1-ERα pathway in male animals (Meitzen et al. 2017; Hohoff et al. 2019), the missing *Zdhhc7* in KO mice could possibly lead to a reduced transmission and impaired LTP (Meitzen et al. 2017; Hohoff et al. 2019). In females, CAV1 and ZDHHC7 are abundantly available in neonatal rat hippocampi, and the levels decrease in adulthood (Meitzen et al. 2017). If these results are transferable to mice, *Zdhhc7*-deficiency would affect females in early life stages. In addition, *Zdhhc7* might influence neonatal organization of the hippocampus in females via rapid estradiol actions of membrane ER-mGluR coupling (Meitzen et al. 2017). These estradiol actions regulate phosphorylation of the cAMP response element-binding (CREB) protein, which is crucial for the development of normal, female-specific hippocampus organization (Boulware et al. 2005). Therefore, a *Zdhhc7*-deficiency could perhaps lead to LTP impairment in both male and female mice, although it would affect different sex-specific pathways at different stages of life.

In a previous study, Wang et al. (2019) found enhancement of LTP in the hippocampal CA1 region immediately after 30 min of restraint stress via AMPAR trafficking and excitatory synaptic transmission in this region due to glucocorticoid activation. In mice, intra-hippocampal levels of corticosterone, the major glucocorticoid responsible for stress effects (Hill et al. 2008), rose to 240 nM after 15 min of acute stress (Chauveau et al. 2010). Furthermore, in the rat hippocampus, applying 200 nM corticosterone eased AMPAR trafficking and, thereby, enhanced LTP induction (Whitehead et al. 2013). Interestingly, in the current study acute stress improved LTP exclusively in female WTs and KOs compared to control females (Fig. 5c, e), possibly due to new receptor synthesis following the acute stress paradigm.

On the other hand, in the current study acute stress increased LTP in male WTs but not in stressed male KOs (Fig. 5d, f). This might be linked to the structural modification of the hippocampus-mPFC pathway in male KOs (Lynch 2004; Tripathi et al. 2016; Hohoff et al. 2019). Excitatory glutamatergic function and LTP are also based on mGluRs (Lynch 2004), which functionally interact with ERs through pairing with different CAVs (e.g., Boulware et al. 2005; Meitzen et al. 2012; Hohoff et al. 2019). For instance, CAV1 couples mGluR1 with ERα, while CAV3 couples mGluR2 with ERα and ERβ in the hippocampus (Boulware and Mermelstein 2009; Meitzen and Mermelstein 2011). In all cases, ERs are palmitoylated and, for instance, the CAV1-mGluR1-ERα coupling strongly resembles the aforementioned ZDHHC7/CAV1-ERα pathway suggested by Meitzen et al. (2017). Thus, the missing LTP improvement observed in male mutants might be because both ERα and ERβ are involved in the modulation of pre- and postsynaptic glutamatergic signaling (Oberlander and Woolley 2016; Hohoff et al. 2019).

The current study on 11-week-old mice revealed similar results as our recent study (17-week-olds; Hohoff et al. 2019) regarding synaptic plasticity, although there were no impairments in hippocampal connectivity in KO mice and no reduction of anxiety-related behavior in female KOs. As mentioned above, rapid neonatal estradiol actions via ER-mGluR coupling are crucial for female-specific hippocampus organization, while ZDHHC7/CAV1-ERα pathways might play a role in glutamatergic transmission later on. From this perspective, it is quite plausible that the discrepancies between males and females, as well as between KOs and WT, become more apparent with age. Therefore, further analyses with *Zdhhc7* KOs of different age groups are warranted.

Overall, our study revealed for the first time that even a short stressor of 1 h significantly affects gene expression, hippocampal microstructure and synaptic plasticity in a sex-specific manner. It would, therefore, be interesting to examine in a follow-up study the effects of a chronic stress paradigm on sex-specific stress behaviors of *Zdhhc7*-deficient mice.

## Supplementary Information

Below is the link to the electronic supplementary material.

Supplementary file1 (DOCX 3370 kb)

## Data Availability

Data used in the present study are available upon request to the primary contact author.

## References

[CR1] Agarwal A, Zhang M, Trembak-Duff I, Unterbarnscheidt T, Radyushkin K, Dibaj P, Martins de Souza D, Boretius S, Brzózka MM, Steffens H, Berning S, Teng Z, Gummert MN, Tantra M, Guest PC, Willig KI, Frahm J, Hell SW, Bahn S, Rossner MJ, Nave KA, Ehrenreich H, Zhang W, Schwab MH (2014). Dysregulated expression of neuregulin-1 by cortical pyramidal neurons disrupts synaptic plasticity. Cell Rep.

[CR2] Balthazart J, Ball GF (2006). Is brain estradiol a hormone or a neurotransmitter?. Trends Neurosci.

[CR3] Bandler R, Keay KA, Floyd N, Price J (2000). Central circuits mediating patterned autonomic activity during active vs. passive emotional coping. Brain Res Bull.

[CR4] Baudry M, Bi X, Aguirre C (2013). Progesterone-estrogen interactions in synaptic plasticity and neuroprotection. Neuroscience.

[CR5] Bennett MR (2011). The prefrontal-limbic network in depression: a core pathology of synapse regression. Prog Neurobiol.

[CR6] Boulware MI, Mermelstein PG (2009). Membrane estrogen receptors activate metabotropic glutamate receptors to influence nervous system physiology. Steroids.

[CR7] Boulware MI, Weick JP, Becklund BR, Kuo SP, Groth RD, Mermelstein PG (2005). Estradiol activates group I and II metabotropic glutamate receptor signaling, leading to opposing influences on cAMP response element-binding protein. J Neurosci.

[CR8] Brinton RD, Thompson RF, Foy MR, Baudry M, Wang J, Finch CE, Morgan TE, Pike CJ, Mack WJ, Stanczyk FZ, Nilsen J (2008). Progesterone receptors: form and function in brain. Front Neuroendocrinol.

[CR9] Chauveau F, Tronche C, Piérard C, Liscia P, Drouet I, Coutan M, Béracochéa D (2010). Rapid stress-induced corticosterone rise in the hippocampus reverses serial memory retrieval pattern. Hippocampus.

[CR10] Conrad CD (2008). Chronic stress-induced hippocampal vulnerability: the glucocorticoid vulnerability hypothesis. Rev Neurosci.

[CR11] Damasio AR, Grabowski TJ, Bechara A, Damasio H, Ponto LL, Parvizi J, Hichwa RD (2000). Subcortical and cortical brain activity during the feeling of self-generated emotions. Nat Neurosci.

[CR12] Deacon RMJ (2006). Assessing nest building in mice. Nat Protoc.

[CR13] Drzewiecki CM, Willing J, Juraska JM (2016). Synaptic number changes in the medial prefrontal cortex across adolescence in male and female rats: a role for pubertal onset. Synapse.

[CR14] Franklin KBJ, Chudasama Y, Watson C, Paxinos G, Puelles L (2012). Chapter 30—prefrontal cortex. The mouse nervous system.

[CR15] Frye CA (2011). Progesterone attenuates depressive behavior of younger and older adult C57/BL6, wildtype, and progesterone receptor knockout mice. Pharmacol Biochem Behav.

[CR16] Fukata Y, Fukata M (2010). Protein palmitoylation in neuronal development and synaptic plasticity. Nat Rev Neurosci.

[CR17] Georgiou P, Zanos P, Jenne CE, Gould TD (2019). Sex-specific involvement of estrogen receptors in behavioral responses to stress and psychomotor activation. Front Psychiatry.

[CR18] Handley SL, Mithani S (1984). Effects of alpha-adrenoceptor agonists and antagonists in a maze-exploration model of 'fear'-motivated behavior. Naunyn-Schmiedeberg’s Arch Pharmacol.

[CR19] Hemmer K, Zhang M, van Wüllen T, Sakalem M, Tapia N, Baumuratov A (2014). Induced neural stem cells achieve long-term survival and functional integration in the adult mouse brain. Stem Cell Reports.

[CR20] Hill EE, Zack E, Battaglini C, Viru M, Viru A, Hackney AC (2008). Exercise and circulating cortisol levels: the intensity threshold effect. J Endocrinol Invest.

[CR21] Hohoff C, Zhang M, Ambrée O, Kravchenko M, Buschert J, Kerkenberg N, Gorinski N, Abdel Galil D, Schettler C, Vom Werth KL, Wewer MF, Schneider I, Grotegerd D, Wachsmuth L, Faber C, Skryabin BV, Brosius J, Ponimaskin E, Zhang W (2019). Deficiency of the palmitoyl acyltransferase ZDHHC7 impacts brain and behavior of mice in a sex-specific manner. Brain Struct Funct.

[CR22] Kim DH, Kim DY, Kim YC, Jung JW, Lee S, Yoon BH, Cheong JH, Kim YS, Kang SS, Ko KH, Ryu JH (2007). Nodakenin, a coumarin compound, ameliorates scopolamine-induced memory disruption in mice. Life Sci.

[CR23] Kim H., Call T, Carotenuto S, Johnson, R, Ferguson, D (2017) Testing depression in mice: a chronic social defeat stress model. Bio-protocol 7(7): e2203. www.bio-protocol.org/e2203. Accessed 9 June 2020 10.21769/BioProtoc.2203 PMC8410330 34541213

[CR24] Koss WA, Frick KM (2017). Sex differences in hippocampal function. J Neurosci Res.

[CR25] Kreher BW, Hennig J, Il’yasov KA (2006) DTI&FiberTools: a complete toolbox for DTI calculation, fiber tracking and combined evaluation. In: Proceeding of ISMRM 14t international scientific meeting Seattle, USA

[CR26] Kudwa AE, Harada N, Honda SI, Rissman EF (2009). Regulation of progestin receptors in medial amygdala: estradiol, phytoestrogens and sex. Physiol Behav.

[CR27] Kumar S, Black SJ, Hultman R, Szabo ST, DeMaio KD, Du J, Katz BM, Feng G, Covington HE, Dzirasa K (2013). Cortical control of affective networks. J Neurosci.

[CR28] Lehmann ML, Weigel TK, Elkahloun AG, Herkenham M (2017). Chronic social defeat reduces myelination in the mouse medial prefrontal cortex. Sci Rep.

[CR29] Livak KJ, Schmittgen TD (2001). Analysis of relative gene expression data using real-time quantitative PCR and the 2(-Delta Delta C(T))method. Methods.

[CR30] Lynch MA (2004). Long-term potentiation and memory. Physiol Rev.

[CR31] Mackintosh JH, Berry AJ (1981). Behaviour of the House Mouse. The proceedings of symposium on biology of the house mouse.

[CR32] Marrocco J, McEwen BS (2016). Sex in the brain: hormones and sex differences. Dialogues Clin Neurosci.

[CR33] Marrocco J, Petty GH, Ríos MB, Gray JD, Kogan JF, Waters EM, Schmidt EF, Lee FS, McEwen BS (2017). A sexually dimorphic pre-stressed translational signature in CA3 pyramidal neurons of BDNF Val66Met mice. Nat Commun.

[CR34] Meitzen J, Mermelstein PG (2011). Estrogen receptors stimulate brain region specific metabotropic glutamate receptors to rapidly initiate signal transduction pathways. J Chem Neuroanat.

[CR35] Meitzen J, Grove DD, Mermelstein PG (2012). The organizational and aromatization hypotheses apply to rapid, nonclassical hormone action: neonatal masculinization eliminates rapid estradiol action in female hippocampal neurons. Endocrinology.

[CR36] Meitzen J, Luoma JI, Boulware MI, Hedges VL, Peterson BM, Tuomela K, Britson KA, Mermelstein PG (2013). Palmitoylation of estrogen receptors is essential for neuronal membrane signaling. Endocrinology.

[CR37] Meitzen J, Britson KA, Tuomela K, Mermelstein PG (2017). The expression of select genes necessary for membrane-associated estrogen receptor signaling differ by sex in adult rat hippocampus. Steroids.

[CR38] Naumenko VS, Ponimaskin E (2018). Palmitoylation as a functional regulator of neurotransmitter receptors. Neur Plast.

[CR39] Norman AW, Mizwicki MT, Norman DP (2004). Steroid-hormone rapid actions, membrane receptors and a conformational ensemble model. Nat Rev Drug Discov.

[CR40] Oberlander JG, Woolley CS (2016). 17β-Estradiol acutely potentiates glutamatergic synaptic transmission in the hippocampus through distinct mechanisms in males and females. J Neurosci.

[CR41] Oh SW, Harris JA, Ng L, Winslow B, Cain N, Mihalas S, Wang Q, Lau C, Kuan L, Henry AM, Mortrud MT, Ouellette B, Nguyen TN, Sorensen SA, Slaughterbeck CR, Wakeman W, Li Y, Feng D, Ho A, Nicholas E, Hirokawa KE, Bohn P, Joines KM, Peng H, Hawrylycz MJ, Phillips JW, Hohmann JG, Wohnoutka P, Gerfen CR, Koch C, Bernard A, Dang C, Jones AR, Zeng H (2014). A mesoscale connectome of the mouse brain. Nature.

[CR42] Ooishi Y, Kawato S, Hojo Y, Hatanaka Y, Higo S, Murakami G, Komatsuzaki Y, Ogiue-Ikeda M, Kimoto T, Mukai H (2012). Modulation of synaptic plasticity in the hippocampus by hippocampus-derived estrogen and androgen. J Steroid Biochem Mol Biol.

[CR43] Oyola MG, Handa RJ (2017). Hypothalamic–pituitary–adrenal and hypothalamic–pituitary–gonadal axes: sex differences in regulation of stress responsivity. Stress.

[CR44] Pedram A, Razandi M, Deschenes RJ, Levin ER (2012). DHHC-7 and -21 are palmitoylacyltransferases for sex steroid receptors. MolBiol Cell.

[CR45] Ponimaskin E, Dityateva G, Ruonala MO, Fukata M, Fukata Y, Kobe F, Wouters FS, Delling M, Bredt DS, Schachner M, Dityatev A (2008). Fibroblast growth factor-regulated palmitoylation of the neural cell adhesion molecule determines neuronal morphogenesis. J Neurosci.

[CR46] Rathenberg J, Kittler JT, Moss SJ (2004). Palmitoylation regulates the clustering and cell surface stability of GABAA receptors. Mol Cell Neurosci.

[CR47] Saffari R, Teng Z, Zhang M, Kravchenko M, Hohoff C, Ambrée O, Zhang W (2016). NPY+-, but not PV+-GABAergic neurons mediated long-range inhibition from infra-to prelimbic cortex. Transl Psychiatry.

[CR48] Sapolsky RM (2001). Depression, antidepressants, and the shrinking hippocampus. Proc Natl Acad Sci USA.

[CR49] Seney ML, Sibille E (2014). Sex differences in mood disorders: perspectives from humans and rodent models. Biol Sex Differ.

[CR50] Shipston MJ (2011). Ion channel regulation by protein palmitoylation. J Biol Chem.

[CR51] Shors TJ, Chua C, Falduto J (2001). Sex differences and opposite effects of stress on dendritic spine density in the male versus female hippocampus. J Neurosci.

[CR52] Shors TJ, Falduto J, Leuner B (2004). The opposite effects of stress on dendritic spines in male vs. female rats are NMDA receptor-dependent. Eur J Neurosci.

[CR53] Suzuki H, Barros RP, Sugiyama N, Krishnan V, Yaden BC, Kim HJ, Warner M, Gustafsson JÅ (2013). Involvement of estrogen receptor β in maintenance of serotonergic neurons of the dorsal raphe. Mol Psychiatry.

[CR54] Tripathi A, Schenker E, Spedding M, Jay TM (2016). The hippocampal to prefrontal cortex circuit in mice: a promising electrophysiological signature in models for psychiatric disorders. Brain Struct Funct.

[CR55] Uylings HB, Groenewegen HJ, Kolb B (2003). Do rats have a prefrontal cortex?. Behav Brain Res.

[CR56] Walf AA, Frye CA (2007). The use of the elevated plus maze as an assay of anxiety-related behavior in rodents. Nat Protoc.

[CR57] Walf AA, Koonce CJ, Frye CA (2009). Adult female wildtype, but not oestrogen receptor beta knockout, mice have decreased depression-like behaviour during pro-oestrus and following administration of oestradiol or diarylpropionitrile. J Psychopharmacol.

[CR58] Wang M, Ramasamy VS, Samidurai M, Jo J (2019). Acute restraint stress reverses impaired LTP in the hippocampal CA1 region in mouse models of Alzheimer's disease. Sci Rep.

[CR59] Wehr MC, Hinrichs W, Brzózka MM, Unterbarnscheidt T, Herholt A, Wintgens JP, Papiol S, Soto-Bernardini MC, Kravchenko M, Zhang M, Nave KA, Wichert SP, Falkai P, Zhang W, Schwab MH, Rossner MJ (2017). Spironolactone is an antagonist of NRG 1 -ERBB 4 signaling and schizophrenia-relevant endophenotypes in mice. EMBO Mol Med.

[CR60] Whitehead G, Jo J, Hogg EL, Piers T, Kim DH, Seaton G, Seok H, Bru-Mercier G, Son GH, Regan P, Hildebrandt L, Waite E, Kim BC, Kerrigan TL, Kim K, Whitcomb DJ, Collingridge GL, Lightman SL, Cho K (2013). Acute stress causes rapid synaptic insertion of Ca2+ -permeable AMPA receptors to facilitate long-term potentiation in the hippocampus. Brain.

[CR61] Zagni E, Simoni L, Colombo D (2016). Sex and gender differences in central nervous system-related disorders. Neurosci J.

