# Supplementary material for: Acute stress reveals different impacts in male and female Zdhhc7-deficient mice
Source: Brain Struct Funct. 2021 Apr 20;226(5):1613–26. doi: 10.1007/s00429-021-02275-y (PMC8096773; doi:10.1007/s00429-021-02275-y)
Supplement: Supplementary file 1 — Supplementary file1 (DOCX 3370 kb) [file 429_2021_2275_MOESM1_ESM.docx]

**Acute stress reveals different impacts in male and female *Zdhhc7*-deficient mice**

Nicole Kerkenberg^1,2#^, Christa Hohoff^1^, Mingyue Zhang^1^, Ilona Lang^1,2^ Christiane Schettler^1^, Evgeni Ponimaskin^3^, Lydia Wachsmuth^4^, Cornelius Faber^2,4^, Bernhard T. Baune^1,2,5,6^, Weiqi Zhang^1,2^

^1^Department of Mental Health, University of Münster, Münster, Germany

^2^Otto Creutzfeldt Center for Cognitive and Behavioral Neuroscience, University of Münster, Münster, Germany

^3^Cellular Neurophysiology, Hannover Medical School, Hannover, Germany

^4^Clinic of Radiology, University of Münster, Münster, Germany

^5^Department of Psychiatry, Melbourne Medical School, University of Melbourne, Melbourne, Victoria, Australia

^6^Florey Institute for Neuroscience and Mental Health, University of Melbourne, Melbourne, Victoria, Australia

^#^ **Corresponding author:**

Nicole Kerkenberg; Email: [Nicole.kerkenberg@ukmuenster.de](mailto:Nicole.kerkenberg@ukmuenster.de)

Weiqi Zhang; Email: [wzhang@uni-muenster.de](mailto:wzhang@uni-muenster.de)

**Electronic Supplementary Material**

**Suppl. Figures**


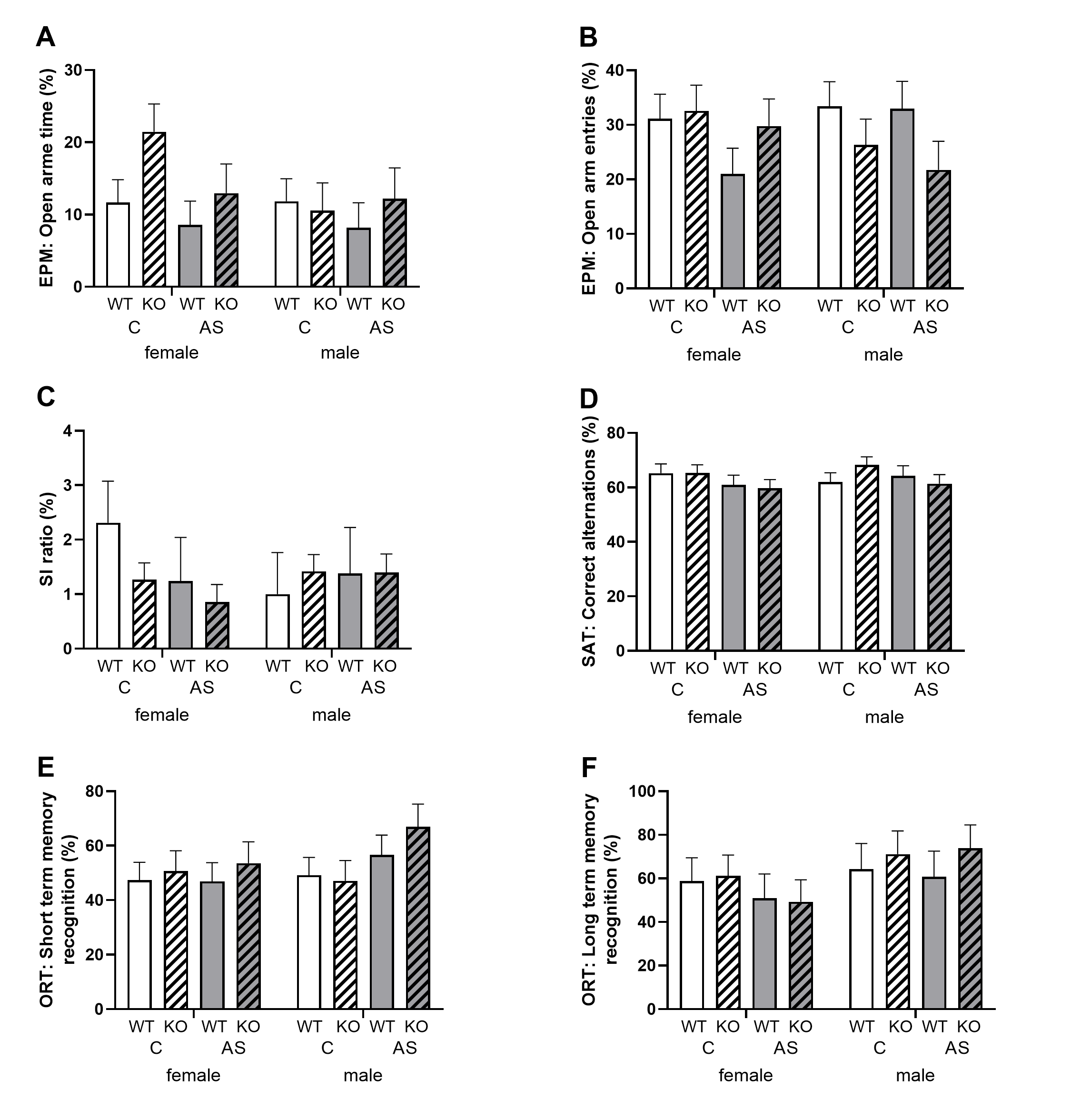


**Suppl. Fig. 1** Effects of *Zdhhc7*-deficiency and acute stress on anxiety-related behavior, sociability, spatial working, and object recognition memory.

*Zdhhc7*-KO and -WT mice of both sexes and conditions (control (C) and acute stress (AS)) were analyzed in the elevated plus maze test (EPM) (**A-B**), social interaction test (SI) (**C**), spontaneous alternation test (SAT) (**D**), and object recognition test (ORT) (**E-F**). No significant effects regarding genotype, stress or sex were found in any of the measured parameters. Bars represent group means (± SEM) and sample sizes were as follows: control female littermates, 11; control male littermates, 11; stressed female littermates, 10; and stressed male littermates, 9.


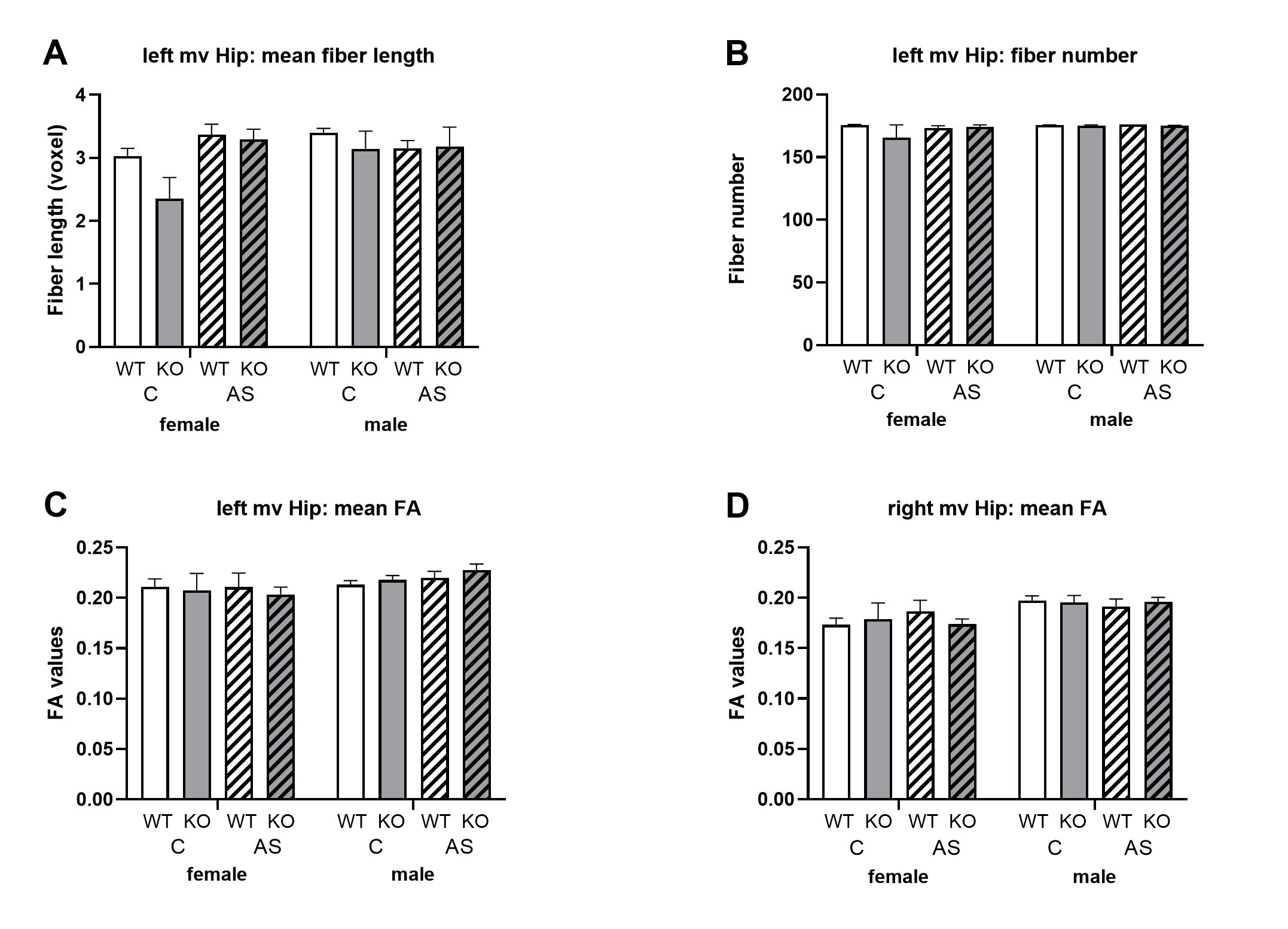


**Suppl. Fig. 2** Effects of *Zdhhc7*-deficiency and acute stress on left and right hippocampal fiber structures.

*Zdhhc7*-KO and -WT mice of both sexes and conditions (control (C) and acute stress (AS)) were analyzed with respect to DTI&Fiber Tool-based images to obtain mean fiber length (**A**), fiber number (**B**), and mean FA (**C-D**) in the medioventral hippocampal CA region. No significant effects regarding genotype, stress or sex were found in any of the measured parameters. Bars represent group means (± SEM) and sample sizes were as follows: control female littermates, 4; control male littermates, 4; stressed female littermates, 4; and stressed male littermates, 4.





**Suppl. Fig. 3** Effects of *Zdhhc7*-deficiency and acute stress on expression of candidate genes in the left (**A** – **F**) and right (**G** – **L**) mPFC.

*Zdhhc7*-KO and -WT mice of both sexes and conditions (control (C) and acute stress (AS)) were analyzed regarding gene expression of estrogen receptor α (*Esr1)* (**A**), estrogen receptor β (*Esr2)* (**B**), progesterone receptor (*Pgr)* (**C**), androgen receptor (*Ar)* (**D**), Caveolin 1 (*Cav1*) (**E**), and caveolin 3 (*Cav3*) (**F**) in the left mPFC as well as of *Esr1* (**G**), *Esr2* (**H**), *Pgr* (**I**), *Ar* (**J**), *Cav1* (**K**), and *Cav3* (**L**) in the right mPFC. Values were calculated as described in the methods. The lower the gene expression, the higher the ΔC_t_ value. Effects regarding stress were found for *Esr1* (**A**), *Esr2* (**B**), and *Ar* (**D**), while sex effects were found for *Esr1* (**A**), *Esr2* (**B**), *Ar* (**D**), and *Pgr* (**I**) Bars represent group means (± SEM) and sample sizes were as follows: control female littermates, 4; control male littermates, 5; stressed female littermates, 6; and stressed male littermates, 5.


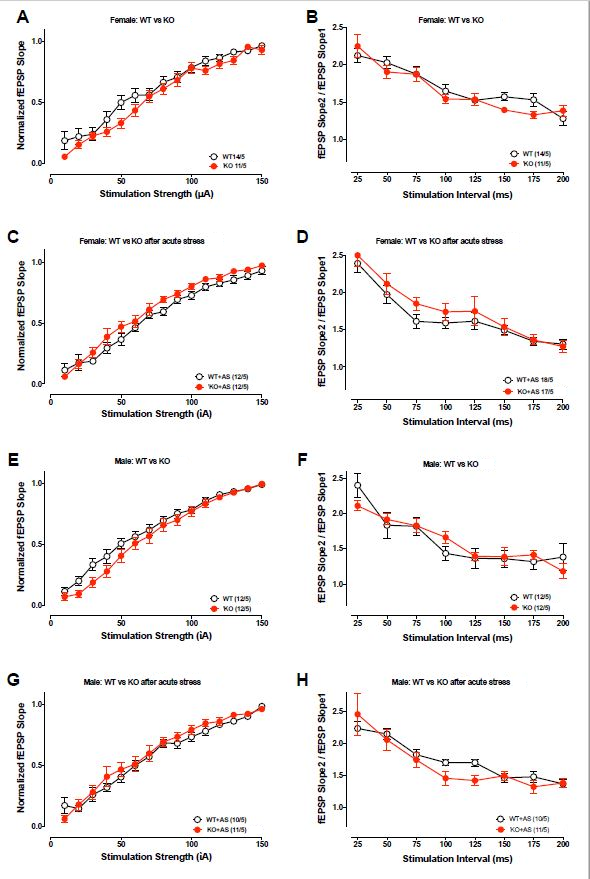


**Suppl. Fig. 5** Effects of *Zdhhc7*-deficiency and acute stress on synaptic plasticity.

(**A**, **C**) Input-output curves, as a measure of baseline excitatory synaptic transmission (depicted as fEPSP slope plotted against the stimulation strength), were not altered in the hippocampal CA1 region of female WT and KO mice with and without acute stress (AS). (**B**, **D**) Paired-pulse ratios were not significantly changed in the hippocampal CA1 region of female WT and KO mice with and without acute stress. (**E**, **G**) Input-output curves, as a measure of baseline excitatory synaptic transmission (depicted as fEPSP slope plotted against the stimulation strength), were not altered in the hippocampal CA1 region of male WT and KO with and without acute stress. (**F**, **H**) Paired-pulse ratios were not significantly changed in the hippocampal CA1 region of male WT and KO mice with and without acute stress. Dots represent group means (± SEM), with the bottom numbers indicating sample size per group (n/N: recordings/total number of animals).
